# Structural Characteristics of the 5′-Terminal Region of Mouse p53 mRNA and Identification of Proteins That Bind to This mRNA Region

**DOI:** 10.3390/ijms23179709

**Published:** 2022-08-26

**Authors:** Joanna Szpotkowska, Kamil Szpotkowski, Jerzy Ciesiołka

**Affiliations:** 1 Institute of Bioorganic Chemistry, Polish Academy of Sciences, 61-704 Poznan, Poland; 2 Department of Gene Expression, Faculty of Biology, Institute of Molecular Biology and Biotechnology, Adam Mickiewicz University Poznan, 61-614 Poznan, Poland

**Keywords:** mouse p53 mRNA, 5′ non-coding region, RNA structure, RNA-assisted affinity chromatography, hnRNP K, PCBP2

## Abstract

A mouse model has often been used in studies of *p53* gene expression. Detailed interpretation of functional studies is, however, hampered by insufficient knowledge of the impact of mouse p53 mRNA’s structure and its interactions with proteins in the translation process. In particular, the 5′-terminal region of mouse p53 mRNA is an important region which takes part in the regulation of the synthesis of p53 protein and its N-truncated isoform Δ41p53. In this work, the spatial folding of the 5′-terminal region of mouse p53 mRNA and its selected sub-fragments was proposed based on the results of the SAXS method and the RNAComposer program. Subsequently, RNA-assisted affinity chromatography was used to identify proteins present in mouse fibroblast cell lysates that are able to bind the RNA oligomer, which corresponds to the 5′-terminal region of mouse p53 mRNA. Possible sites to which the selected, identified proteins can bind were proposed. Interestingly, most of these binding sites coincide with the sites determined as accessible to hybridization of complementary oligonucleotides. Finally, the high binding affinity of hnRNP K and PCBP2 to the 5′-terminal region of mouse p53 mRNA was confirmed and their possible binding sites were proposed.

## 1. Introduction

p53 plays an important role in maintaining cell homeostasis [1,2,3]. This protein is a transcription factor which regulates the expression of many genes, especially in stress conditions, under which the p53 level is elevated. In a healthy cell, the p53 level is low due to its ubiquitination by Mdm2 and then its degradation. In response to stress conditions, p53 is phosphorylated to prevent the Mdm2–p53 interaction and the p53 level gets higher [1,2,3]. Besides the full-length p53, there are several isoforms of this protein in the cell whose expression results from the usage of various transcription promotors, alternative splicing and various translation initiation sites [4,5,6,7]. The isoforms show tissue specificity, and their expression levels vary depending on tissue type. Importantly, it has been shown that disturbance of the p53 isoforms’ expression plays a very important role in tumorigenesis [8,9,10], cell differentiation [11] and cell responses to pathogens [12].

It has been shown that the expression of human *TP53* is regulated at several levels, and several proteins are involved in this regulation. Protein factors that bind to p53 mRNA are potentially involved in translation regulation. Regulation at the level of p53 mRNA translation determines the amounts of synthetized p53 protein and its isoforms [13,14,15,16]. The 5′-terminal region of p53 mRNA plays a crucial role in translation initiation, particularly when the p53 level must be quickly elevated in response to stress conditions. It has been hypothesized that an increase in translation efficiency in such conditions results from the activity of two internal ribosomal entry sites (IRESes) [17,18]. Recently, we have demonstrated that changes introduced to the 5′-terminal region of p53 mRNA greatly influence ribosomal scanning and p53 translation efficiency [19]. This region was also targeted by antisense oligonucleotides which modulated the expression level of p53 protein [20,21]. We have shown that the 5′-terminus of p53 mRNA folds into several characteristic structural motifs [22,23]. These motifs presumably act as docking platforms for numerous regulatory factors, such as proteins [16,24,25] or antisense transcripts [26,27]. Recently, we have shown that two poly(C)-binding proteins, hnRNP K and PCBP2, can bind to the 5′-terminal region of human p53 mRNA, influencing the synthesis of p53 [28,29]. All the above data support the notion that folding of the 5′-terminal region of p53 mRNA and protein factors interacting with this region greatly influence the expression of *TP53*.

The mouse has often been used as a model organism in functional studies of p53, and the most spectacular observation derived from this model is that mice devoid of the *Trp53* gene are highly tumor prone [30]. Recent review papers discussed in detail the contributions of studies on mouse models to the knowledge of p53 expression and p53’s novel roles in promoting tissue homeostasis, and cell invasion and metastasis [31]. However, it has to be noted that there is still very limited information on mouse p53 mRNA transcripts, their translation process and their regulation. In our recent study, we focused on the 5′-terminus of mouse p53 mRNA in order to get an insight into its role in the regulation of p53 protein synthesis [32]. Transcription initiation sites for *Trp53* gene were determined by the 5′RACE method. Subsequently, secondary structure models of the 5′-terminal regions of the most abundant and the longest mouse transcripts were determined by experimental probing in vitro. A comparison of the newly proposed secondary structures of the 5′-termini of two mouse mRNAs with conservation of the nucleotide sequences of these regions in various mammals revealed several conserved structural motifs that may perform the same functions. It has been suggested that these motifs may bind regulatory proteins or antisense oligonucleotides; however, detailed information regarding their involvement in the binding is still missing [32].

Here we describe the results of a comprehensive study of the 5′-terminus of the mouse’s most abundant p53 mRNA transcript concerning its tertiary structure and protein factors interacting with this region. For structural studies, we used small-angle X-ray scattering (SAXS), which has proven to be a powerful method to study, in solution, tertiary structures of RNA, proteins, RNA–protein complexes or disordered proteins [33,34]. The obtained low-resolution structures of the full-length 5′-terminal region of p53 mRNA and its selected fragments corresponding to defined structural motifs were compared to 3D RNA models predicted by RNAComposer [35,36]. Subsequently, RNA-centric affinity chromatography combined with mass spectrometry analysis was used [37,38] to identify proteins interacting with this mRNA region. In a similar affinity chromatography experiment performed for human cells, we identified a set of proteins present in the top candidates’ group. For selected proteins, their predicted binding sites were displayed on the secondary structure model of the 5′-terminal region of mouse p53 mRNA. The predicted sites were also compared with the sites in the RNA that were determined as accessible to hybridization of complementary oligonucleotides. Finally, high binding affinity of human hnRNP K and PCBP2 to the 5′-terminal region of mouse p53 mRNA was confirmed, and their possible binding sites were proposed.

## 2. Results

### 2.1. Tertiary Structure of the 5′-Terminal Region of Mouse p53 mRNA

**Figure 1:**
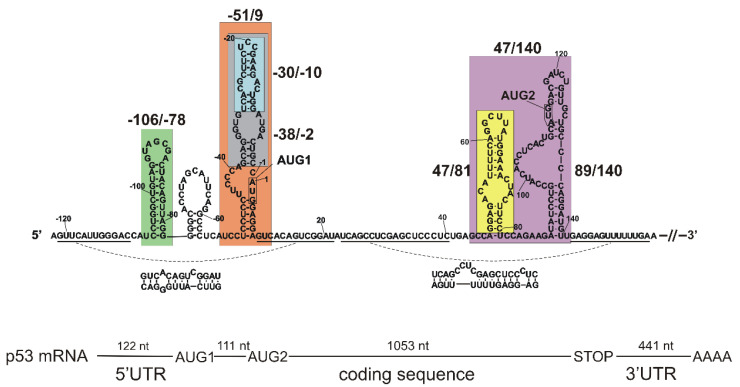
Secondary structure model of the 5′-termini of mouse p53 mRNA with the analyzed elements indicated. The displayed structure consists of the first 278 nucleotides of p53 mRNA and spans the 5′ non-coding region of 122 nucleotides and a part of the coding sequence with the AUG2 initiation codon. Structural elements marked by color boxes were synthetized as separate RNA oligomers. The RNA oligomers are denoted by the numbers in the mRNA sequence of the first and the last nucleotide of each oligomer as follows: -106/-78 (green box), -51/9 (orange box), -30/-10 (blue box), -38/-2 (gray box), 47/140 (violet box), 47/81 (yellow box) and 89/140 (hairpin within a violet box, not marked by separate box). Schematic representation of the full-length mouse p53 mRNA is also shown in the figure.

A secondary structure model of the 5′-terminal region of mouse p53 mRNA has already been proposed based on the results of extensive biochemical mapping studies [32]. In order to characterize spatial folding of this mRNA region, we synthesized a few of its sub-fragments, i.e., the following RNAs: -106/-78, -51/9, -30/-10. -38/-2, 47/81, 47/140 and 89/140 (Figure 1, Table S1). These RNAs correspond to the defined structural motifs of the secondary structure of the 5′-terminal region. Additionally, RNA-122/201 was synthetized, spanning the entire 122-nucleotide-long 5′ non-coding region and a 201-nucleotide-long stretch of the p53 mRNA coding sequence. The oligomers were subjected to a denaturation–renaturation procedure, and finally were suspended in the buffer: 40 mM Tris pH 7.5, 130 mM KCl, 0.5 mM EDTA and 5 mM MgCl_2_. In some experiments, a lower concentration of Mg^2+^ ions was used: 0 or 2.5 mM. Subsequently, the oligomers’ structures were analyzed in solution using the SAXS technique. In the resulting SAXS-derived ab initio envelopes structural models of the oligomers were embedded, which were proposed by the RNAComposer program [35,36].

#### 2.1.1. Hairpin Domain with AUG1 Initiation Codon

**Figure 2:**
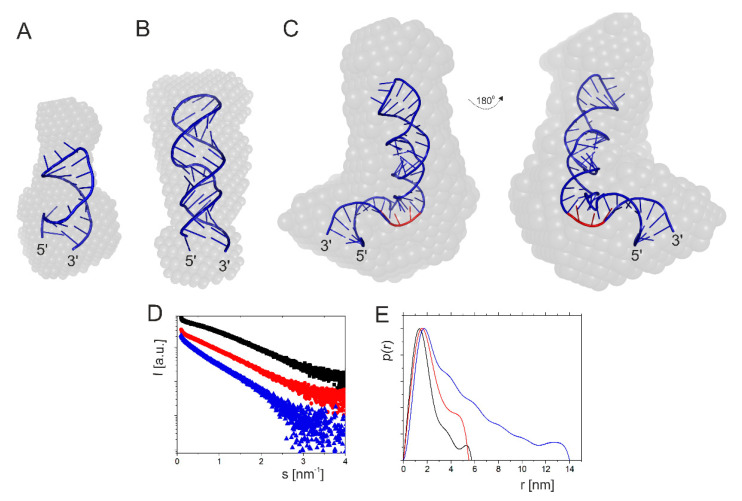
Structural analysis of mRNA fragments: RNA-30/-10 (**A**), RNA-38/-2 (**B**) and RNA-51/9 (**C**). Panels A, B and C present SAXS-derived ab initio envelopes obtained using DAMMIF algorithm superimposed with tertiary structures generated by RNAComposer. The AUG1 initiation codon is marked in red. (**D**) Experimental SAXS curves in the range of vector s from 0.0088 nm^−1^ to 4 nm^−1^. (**E**) Corresponding pair distance distribution function shows the elongated shapes of analyzed fragments of p53 mRNA; these shapes are typical of polyanions in a polar solution: RNA-30/-10—black, RNA-38/-2—red, RNA-51/9—blue.

First, we analyzed the structure of RNA-51/9, which is a 60-nucleotide-long imperfect hairpin domain with AUG1 initiation codon for the full-length p53 protein (Figure 2). The hairpin plays a crucial role in translation initiation. It has earlier been demonstrated for human p53 mRNA that the introduction of structural changes in the hairpin domain possessing AUG1 greatly influences p53 translation efficiency [19].

Analysis of the SAXS data (Table 1) and the linearity of the Guinier region confirmed that the sample of RNA-51/9 was monodisperse. The radius of gyration (R_g_) was calculated in two different ways: from the Guinier plot and from the pair distribution function [P(r)] calculated as the Fourier transform of the scattering curve. The obtained values were 3.87 and 4.10 nm, respectively. The maximum diameter in the particle D_max_ estimated from the pair distribution function was 16.21 nm. The spatial RNA model predicted by the RNAComposer program fits well to the experimental data (χ^2^ = 1.9) and the theoretical and experimental RNA volumes, which were 19,051 and 21,908 nm^3^, respectively. Moreover, the SAXS-derived ab initio envelope and the theoretical RNA model have very similar L-shaped structures (Figure 2C).

In order to check how the shortening of the RNA-51/9 domain correlates with its expected structural and thermodynamic properties, two additional RNAs were analyzed: a 37-nucleotide-long RNA fragment spanning nucleotides G(-38) and C(-2), and a shorter, 21-nucleotide-long RNA oligomer corresponding to the region between U(-30) and G(-10) (Figure 1 and Figure 2A,B). Analysis of the scattering curve of RNA-38/-2 disclosed R_g_ values of 1.96 and 1.94 nm for the Guinier- and P(r)-derived R_g_ values (Table 1). The calculated D_max_ value was 6.32 nm. Theoretical data obtained on the basis of the computationally generated model were R_g_ of 1.74 nm and D_max_ of 6.14 nm, so the fit of the models seemed to be exceptionally good. Very similar data were obtained for RNA-30/-10. The calculated R_g_ values were 1.46 nm (Guinier) and 1.5 nm (P(r)), and D_max_ was 5.07, whereas theoretical data yielded R_g_ of 1.27 nm and D_max_ of 4.17 nm. In this case, we also observed a very good fit of the model. Comparison of the ab initio envelopes of RNAs: -51/9, -38/-2 and -30/-10 reveals that although the initial, longest fragment was shortened by 23 and 39 nucleotides, this did not lead to significant structural rearrangements of its sub-fragments (Figure 2). Thus, it turns out that the ab initio envelope for RNA-30/-10 fits well in the envelope of RNA-38/-2, which in turn matches a larger part of the L-shaped envelope of RNA-51/9.

#### 2.1.2. Two-Hairpins Domain with AUG2 Initiation Codon

Next, we focused on a highly evolutionarily conserved mRNA region spanning nucleotides A47 and U140 (Figure 1). This region contains two stable hairpin motifs which are crucial for mRNA functioning. The first hairpin is analogous to that responsible for the binding of E3 ubiquitin ligase Mdm2 in human p53 mRNA [39], and the Mdm2 protein is the main known p53 negative regulator in the cell. In human cells, Mdm2 can interact both with the p53 protein and with p53 mRNA. Thus, p53 and Mdm2 regulate each other by forming a negative-feedback loop [39].

We have earlier observed that in mouse p53 mRNA the presence of an Mdm2-binding hairpin is not indisputable, since four nucleotides, C45, G51, C82 and C83, are different than those present at the corresponding positions in human p53 mRNA, nucleotides U, A, G and A, respectively [32]. This causes shortening of the hairpin stem by two base pairs and the formation of a non-canonical GU base pair closing the internal bulge. In silico analysis revealed that for this region of mouse p53 mRNA, two small hairpins composed of 26 and 13 nucleotides are thermodynamically preferred. However, mapping of RNA accessibility to hybridization with DNA semi-random libraries and RNase H cleavage [40,41] has shown that a single hairpin motif similar to that of the human Mdm2-binding hairpin is the dominant fold of this region [32]. Therefore, in this work we assumed a secondary structure of domain 47/140 consisting of two hairpins, one which potentially can bind to the Mdm2 protein and the other in which the AUG2 initiation codon is embedded.

**Figure 3:**
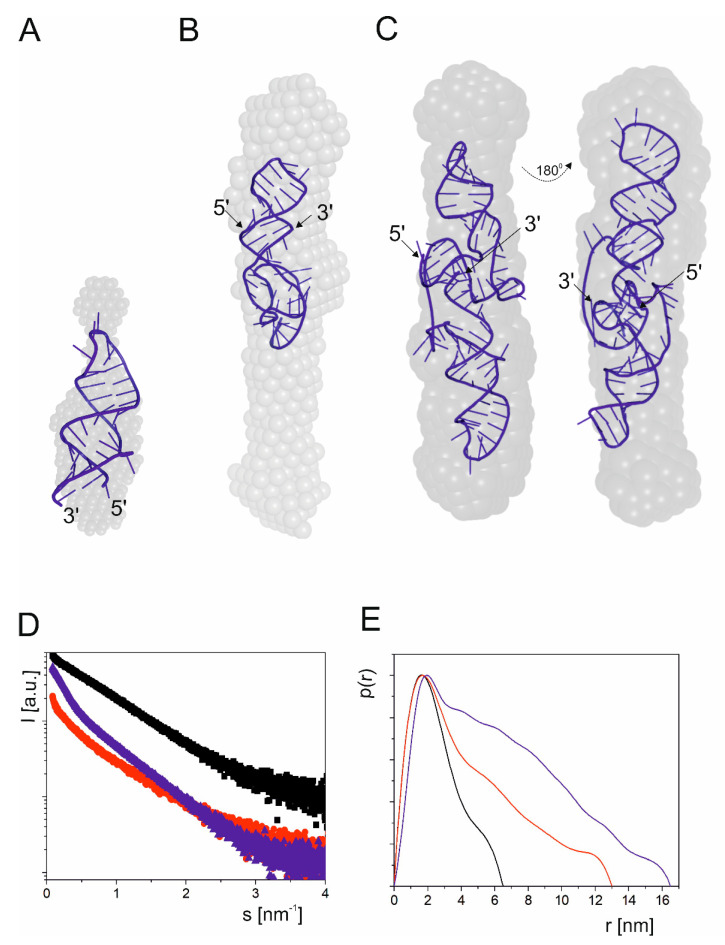
Structural analysis of mRNA fragments: RNA47/81 (**A**), RNA89/140 (**B**) and RNA47/140 (**C**). SAXS-derived ab initio envelopes are superimposed with tertiary structures generated by the RNAComposer program. Experimental SAXS curves (**D**) and corresponding pair distance distribution functions (**E**) are shown for: RNA47/81—black, RNA89/140—red and RNA47/140—blue.

Three fragments of mouse p53 mRNA were synthetized: RNA47/81, RNA89/140 and RNA47/140 (Figure 1). The first fragment, RNA47/81, was 35 nucleotides in length, and it was an analogue of the human hairpin motif that binds to the Mdm2 protein. The SAXS-derived R_g_ values for this RNA were 2.099 nm (Guinier) and 2.078 nm (P(r)), and its D_max_ was 7.1 nm (Table 1). The next fragment, RNA89/140, was a 52-nucleotide-long hairpin with the AUG2 initiation codon for p53 protein isoform Δ41p53, whose human counterpart Δ40p53 may influence cells viability via controlling a full-length p53 protein [42]. The fragment RNA89/140 had a large bulge of 13 nucleotides, which potentially could serve as an easily accessible platform for the binding of protein factors. Inspection of the scattering curve of RNA89/140 revealed R_g_ values of 4.01 and 4.34 nm for Guinier- and P(r)-derived R_g_ values, and the calculated D_max_ value was 17.65 (Table 1). Finally, the RNA47/140 fragment consisted of both above-mentioned hairpins, and it spanned nucleotides A47 and U140. The SAXS-derived data for RNA47/140 revealed Guinier- and P(r) values of 6.02 and 6.17 nm, respectively, and the calculated D_max_ value was 26.88 nm. The ab initio envelope reconstructions revealed an elongated shape for this domain (Figure 3). Subsequently, we generated a tertiary structure model of RNA47/140 by means of the RNAComposer program. Unexpectedly, when running the program in the default interactive mode, we obtained a U-shaped structure which did not fit to the elongated shape of the ab initio envelope. The U shape of the model is a consequence of the presence of a flexible, 7-nucleotide-long single-stranded RNA stretch, which bends the structure in the middle. The elongated shape of the tertiary structure model of RNA47/140 which fits well to the ab initio envelope was generated running the RNAComposer program in the batch mode (Figure 3).

Folding of the Mdm2-binding RNA region deserves special attention. It has earlier been shown that in human p53 mRNA, this region forms a hairpin structure whose tertiary structure is important to maintain its interaction with an oligonucleotide binding pocket in Mdm2 [39]. Based on our results showing that a very similar hairpin motif is present in mouse p53 mRNA, we decided to compare tertiary structure models of human and mouse Mdm2-binding hairpins in order to verify if its function may be preserved between species. To this end, we used the SETTER web server [43], a good tool for accurate and fast comparison of RNA 3D structure models. The calculated RMSD (root mean square difference) was 1.452 Å, and *p*-value and S-distance were only 0.027 and 0.208, respectively. RMSD is a commonly used value to assess similarity of structures: the lower the RMSD value, the more similar the compared structures [44]. S-distance represents overall alignment quality, and *p*-value indicates statistical significance of alignment scores. The lower the *p*-value, the more statistically significant the S-distance [43]. The obtained results indicate high similarity of the structures; thus, we can conclude that the tertiary structure of Mdm2-binding hairpin is preserved between species, and presumably, its ability to bind Mdm2-protein may remain unchanged. However, more detailed research into single nucleotide changes is needed, since it has been proven that the silent single-point mutation L22L in human p53 mRNA reduces Mdm2 binding [39], and some differences in the nucleotide sequence of the hairpin apical loop between human and mouse are observed.

#### 2.1.3. Influence of Magnesium Ions on the Structure of Short Hairpin Motifs

The formation of stably folded RNA structures requires the presence of cations which play a critical role in reducing the phosphates repulsion and facilitating the folding of RNAs into biologically active forms [45]. It has been shown that both monovalent and divalent cations stabilize nucleic acid structures by nonspecific interactions. However, only Mg^2+^ ions show a unique stabilizing role, as they are located in specific places in several nucleic acid structures [46,47,48].

We used SAXS and circular dichroism (CD) spectroscopy to determine the impact of Mg^2+^ ions on the structure of two fragments of the 5′-terminal region of p53 mRNA. RNA-106/-78 was a 29-nucletide-long oligomer which corresponds to the hairpin motif located close to the 5′ end of p53 mRNA and RNA-30/-10 of 21 nucleotides in length represented the upper part of the large hairpin domain with AUG1 initiation codon (Figure 1). Structural parameters obtained from SAXS data of both fragments are shown in Table S2, and their corresponding low resolution structures are presented in Figure S1. The SAXS data showed that Mg^2+^ ions caused some structural transformations in RNA-106/-78 and RNA-30/-10 which were visible as the radius of gyration and the maximum diameter of particles increased, and also in the overall shapes of the molecules. Structural reorientation of the polynucleotide chain caused by Mg^2+^ ions was visible on the Kratky plot as an increasing maximum.

The CD spectra of the analyzed p53 fragments had a broad positive peak covering the region from 260 to 300 nm, along with negative peaks at about 210 nm and about 240 nm (Figure S2). It has been reported that A-RNA displays characteristic positive peaks between 250 and 280 nm and two negative peaks at 210 and 240 nm. The A-form duplex is mostly defined by a positive peak at 260 nm and negative peaks at 210 and 240 nm [49]. The CD spectrum of RNA-30/-10 is characterized by five positive peaks at 284, 274, 253, 221 and 208 nm and negative peaks at 210, 234 and 307 nm in the buffer with Mg^2+^ ions. In the same conditions, RNA-106/-78 showed four positive maxima at 273, 253, 225 and 210 nm and negative maxima at 216, 234 and 307 nm. CD spectra collected for both these RNAs in the buffer with no Mg^2+^ ions added were characterized by a smaller number of peaks of smaller intensity. In both buffer conditions (with and without Mg^2+^ ions), an increasing temperature caused decay of weak peaks, and a decrease in the intensity of all the peaks was additionally observed. The results of CD spectroscopy showed that Mg^2+^ ions stabilize the structure of the studied RNA, causing structural reorientation of their polynucleotide chains.

### 2.2. A Tertiary Structure Model of the 5′-Terminus of p53 mRNA

The next analyzed p53 mRNA fragment, RNA-122/201, was 323 nucleotides in length, and it spanned the whole 122-nucleotide-long 5′ non-coding region and a 201-nucleotide-long stretch of the coding sequence (Figure 1). It has been shown that the coding sequence located between AUG1 and AUG2 which is present in this fragment is indispensable for the proper folding of the 5′-termini of mouse and human p53 mRNAs. It enables correct formation of a hairpin structure in which translation initiation codon AUG1 is embedded [22,32].

**Figure 4:**
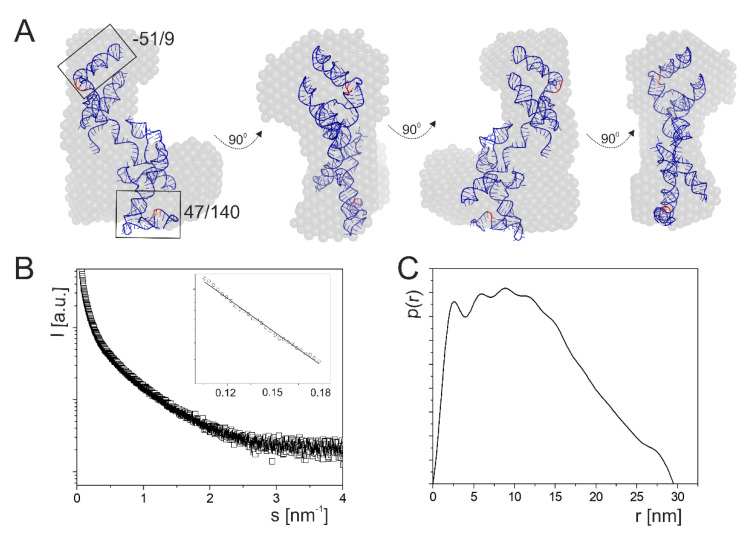
Spatial structure of the 5′-terminal region of mouse p53 mRNA. (**A**) The SAXS-derived ab initio envelope for p53 mRNA fragment RNA-122/201 is superimposed with its tertiary structure generated by RNAComposer. (**B**) Experimental SAXS curve collected for RNA-122/201 and Guinier plot (insert). (**C**) Pair distribution function shows an elongated RNA shape.

Inspection of SAXS data for RNA-122/201 revealed that scattering was characterized by R_g_ of 9.78 and 9.84 nm for Guinier and P(r) analysis, respectively (Table 1). The estimated D_max_ value was 30 nm. The RNA tertiary structure model of RNA-122/201 generated with the use of RNAComposer fits well within the SAXS data, since the χ^2^ value is 2.5 (Figure 4). The SAXS data presented in Table 1 are in good agreement with the theoretical data determined for the RNAComposer model. One essential difference that we observed was between the theoretical and experimental molecule volumes: 101,295 and 126,618 nm^3^. Moreover, for all the described RNA fragments, we can observe that theoretical molecule volume is slightly smaller than the volume determined experimentally (Table 1). This may be due to molecular flexibility in solution that we are not able to observe in theoretical models. Models generated by the RNAComposer program are rigid and do not assume molecular flexibility or movement. Furthermore, the ab initio envelopes determined on the basis of SAXS data not only represent a nucleic acid, but also its surrounding hydration layer [50], which causes an increased molecule volume.

Finally, it has to be noted that in the RNAComposer-generated model, the fragment corresponding to RNA47/140 is clearly folded into a U-shape structure other than the elongated tertiary structure model of isolated RNA47/140, which fits the ab initio envelope. Oppositely, the fragment which corresponds to RNA-51/9 is folded similarly to the isolated RNA or a fragment of the entire 5′-terminal region of p53 mRNA (Figure 4).

### 2.3. Determination of Sites Accessible to Hybridization of Complementary Oligonucleotides within the 5′-Terminal Region of Mouse p53 mRNA

**Figure 5:**
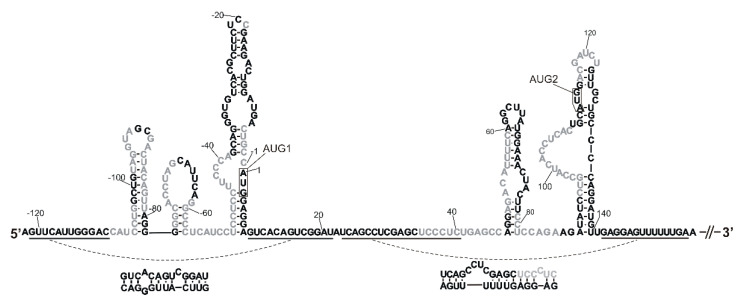
RNA accessibility mapping to oligonucleotide hybridization. Secondary structure model of the 5′-terminal region of mouse p53 mRNA. Nucleotides where at least three oligonucleotides are predicted to bind are marked in grey.

Having determined the tertiary structure model of the 5′-terminal region of mouse p53 mRNA, we decided to assess its accessibility to hybridization of complementary oligonucleotides by using 6-mer semi-random DNA libraries and the RNase H approach [40,41]. Correlation of the predicted positions of hybridizing oligonucleotides with the experimentally determined RNase H cleavages was possible due to the use of oligonucleotide libraries in which the third nucleotides from their 5′ ends were fixed. The full-length p53 mRNA, including the 5′UTR of 122 nucleotides, the entire coding sequence and the 3′UTR, was used as a template for mapping experiments. Accessible sites in the 5′-terminal region of p53 mRNA were identified by primer extension method using radiolabeled primers (Figure S3). The mapping results are displayed on both the tertiary and secondary structure models of the 5′-terminal region of p53 mRNA (Figure 5 and Figure S4). Importantly, in the tertiary structure model of this region generated by RNAComposer, long-range interactions between nucleotide sequences C(-110):G(-122) and G9:U21 and between U23:C40 and G142:156 are present. Moreover, the model has an additional stretch of 42 nucleotides downstream of AUG2 which folds into two small hairpins. Notably, those two hairpins are placed above the non-coding part of the studied RNA, and they do not disturb the overall shape of the structure.

Several accessible regions in the 5′-terminal region of mouse p53 mRNA can be observed (Figure 5 and Figure S4). In the figures, only sites to which at least three predicted oligonucleotides can be bound are marked. The first three accessible regions are close to the 5′ end, C(-109):G(-103), U(-98):A(-93) and G(-90):U(-81); and these regions are located mostly in the C(-106)/G(-78) hairpin. Downstream of the sequence, the accessible regions are G(-76):G(-68) and G(-61):A(-39). These regions are located in hairpin G(-77)/C(-57) and also in the region between hairpin G(-77)/C(-57) and domain U(-50)/A8 and at the bottom-left part of this domain. Thus, oligomer hybridization with the double-stranded regions containing weak base pairs or surrounded by single-stranded RNA regions can be observed. This is consistent with the previous observation that oligomer binding to double-stranded regions is facilitated by structural distortions and weak base pairs in double-stranded regions [20,40,51].

Interestingly, hairpin G(-77)/C(-57) has earlier been described as a binding site for the hnRNP Q protein [16,52]: our accessibility data correspond well with this information. Surprisingly, only one out of seven nucleotides from the region C(-67):G(-61) described earlier as a binding site for hnRNP L [16,53] was determined as accessible to oligomer hybridization. On the other hand, the whole apical loop of this hairpin was cleaved or modified in in vitro secondary structure mapping by means of Pb^2+^-induced cleavage and SHAPE methods [32]. Possibly, the lack of accessibility of region C(-67):A(-62) to oligomer hybridization is a consequence of the involvement of nucleotides of this region in some long-range interactions.

In the case of domain U(-50)/A8, the aforementioned bottom-left part of that domain and the region C(-5):C(-1), immediately preceding the AUG1 initiation codon, are accessible to oligomer hybridization. In both regions, structural distortions are present, which may facilitate oligomer binding. Importantly, the high accessibility of these regions may be beneficial to the binding factors crucial for the regulation of the translation initiation process, as it may regulate the accessibility of the AUG1 codon to the translation machinery. There is also an accessible nucleotide C(-19) in the apical loop of the hairpin motif.

The region U35:C46 adjacent to the Mdm2-binding hairpin A46/U81 on its 5′ side; the regions A50:C59 and C79:A86, spanning bulged nucleotides, a U-track; and three bottom-right nucleotides 79-CCU-81 of the hairpin, are also highly accessible to oligomer hybridization. In the case of hairpin A89/U140, the region C94:C108, mostly bulging out from the hairpin, and the region A116:U122 in the apical loop, namely, the regions between which the AUG2 translation initiation codon is located, are accessible to oligomer hybridization. It is important to note that AUG2 is located between regions accessible to hybridization; therefore, it may serve as a facilitator of AUG2 accession for the factors regulating Δ41p53 synthesis.

When comparing the accessibility of the 5′-terminal region of mouse p53 mRNA to its counterpart in human mRNA [20], we can note similar accessibility in the regions at the bottom part of the large hairpin domain to the AUG1 translation initiation codon. However, in the case of human mRNA, there is great accessibility to the whole Mdm2-binding hairpin, especially in its apical loop region [20], whereas in mouse mRNA the corresponding hairpin is accessible only partially. Importantly, in mouse p53 mRNA, there are regions which are almost not accessible to hybridization, which include the regions of proposed long-range interactions, namely, between G(-121):C(-110) and G9:A20, and between a major part of U23:C40 and G142:A156 (Figure 5 and Figure S4).

### 2.4. Identification of Proteins That Bind to the 5′-Terminal Region of Mouse p53 mRNA

As a next step in our study, we decided to identify proteins which are able to interact with the 5′-terminal region of mouse p53 mRNA. To this end, we used RNA-centric affinity chromatography combined with mass spectrometry analysis [28,37,38]. In the affinity chromatography experiment, we used a cell extract from mouse fibroblast cells, NIH3T3, and as bait, RNA-122/201. The RNA-122/201 was 323 nucleotides in length and consisted of the whole 122-nucleotide-long 5′ non-coding region of p53 mRNA and a 201-nucleotide long stretch of its coding sequence. As a control, we used proteins eluted from agarose beads not coupled with the RNA bait in order to remove any non-specifically bound proteins from further consideration.

The mass spectrometry analysis yielded a list of proteins which are potentially able to interact with the 5′-terminal region of mouse p53 mRNA (Table S3). We focused on the proteins wherein at least two peptides were identified by mass spectrometry. The proteins were classified into four groups (Figure S5). The smallest group, accounting for 2% of the total, consisted of proteins that have earlier been described to interact with mouse p53’s 5′-UTR. The hnRNP Q and hnRNP L proteins [52,53] and ribosomal protein RPL26 [54] were included in this group. Direct interaction of RPL26 with the 5′-UTR of human p53 mRNA was confirmed, and it was proven that overexpression of RPL26 in mouse cell line BaF3 leads to an increased level of p53 [54]. Hence, it can be assumed that the interaction of RPL26 with the 5′-UTR of p53 mRNA is preserved between species. The second group, accounting for 39%, represented proteins classified as translational machinery proteins, such as ribosomal proteins, tRNA ligases or translation initiation factors. The following group, which accounted for 14% of the total, consisted of histone proteins. The presence of histones is understandable, since these proteins are characterized by strong affinity for nucleic acids. The largest group, accounting for 45% of the total, is a group of candidate proteins other than the proteins mentioned above, which are able to interact with the 5′-terminus of p53 mRNA. This group contains proteins with the potential to regulate p53 mRNA translation (Figure S5).

**Figure 6:**
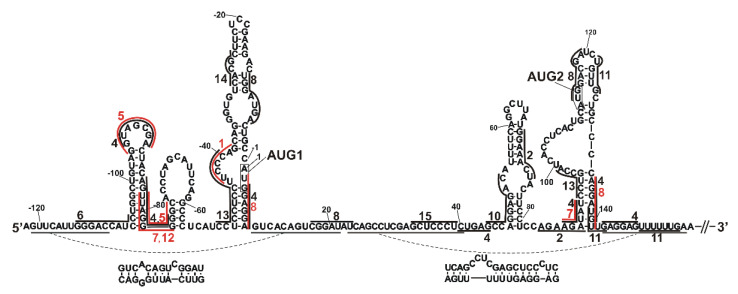
The predicted binding sites for selected protein candidates marked on the secondary structure model of the 5′-terminal region of p53 mRNA. The binding sites are marked with black and red lines along the sequence; red color lines are used to better visualize binding sites in regions where several proteins bind. Binding sites for selected proteins listed in Table 2 were determined based on the RBPmap and ATtRACT databases and on literature information. Predicted binding sites are numbered as follows: 1—nucleolin (UCCCAG), 2—PABPC1 (GGAAACU, AGAAGAU), 4—hnRNP A1 (AGGUAG, GUAGCGA, AGUUAG, UUAGGGG, AGAUAU, CUGAG, GAGGAG, GGAGGA), 5—hnRNP A2/B1 (GGUAGCGA, GUAGCGA, UUAGGGG, AGGGG, GGGGG, GGGGC), 6—PA2G4 (GG(N)_6_CC), 7—hnRNP D0 (UUAGGG, AGAUAU, UUAGGG), 8—G3BP1 (UGGAUGA, UGGAGGA, CGGAUAU, UGGACGA, AGGAUGU), 10—hnRNP A3 (GCCAGGAGAC), 11—FUBP1 (UGUUG, UUUUU, UUUUG), 12—PTBP1 (UUAGGG), 13—PCBP2 (CCUCCUC, CCUUCCC, UUCCC, CCUGCCA), 14—hnRNP K (CACGC), 15—PTBP3 (GCUCCCU). Binding sites for RAN and HMGB1 proteins were not defined, as—according to our knowledge—there has been no literature information on their binding sequences.

In the group of candidates, we identified a set of proteins present in the top 20 positions in the candidates’ group in a similar RNA-centric affinity chromatography experiment that was performed for human cells [28]. We selected 15 proteins with high potential to interact with the 5′-termini of mouse p53 mRNA based on their presence in at least 3 out of 6 analyzed samples from human cells (Table 2). For the selected proteins, their predicted binding sites are displayed on the secondary structure model of 5′-terminal region of mouse p53 mRNA (Figure 6). Binding sites were predicted based on RBPmap [55] and ATtRACT [56] databases and literature information. Where we were unable to find an exactly matching binding sequence for a given protein, we looked for the most similar one. Such prediction was performed for two proteins: nucleolin and polyadenylate binding protein 1 (PABPC1). The original binding sequence for nucleolin is U/GCCCGA [57], and due to the lack of this sequence in the 5′-terminus of mouse p53 mRNA, it was replaced by a highly similar sequence, UCCCAG. For PABPC1, the missing original binding sequence GAAAAC [28] or AG/AAAAAA [55] was replaced by the binding sequences of PABPC1 protein homologs, PABPC5 (GGAAACU, AGAAGAU) and PABPC3 (GGAAACU) (Figure 6). The group of candidates also included proteins which interact—as it has earlier been confirmed—with the human, but not with the mouse, 5′-terminal region of p53 mRNA—in particular, nucleolin, an inhibitor of p53 translation [54], and PTBP1, which regulates IRES-mediated translation of p53 [58,59]. It is worthy of note that in our mass spectrometry analysis, the highest number of peptides was identified for nucleolin (Table 2). Similarly, the highest numbers of peptide spectrum matches for nucleolin have been identified in human cancer cell lines MCF-7, HepG2 and HT-29 in all applied cell conditions [28]. Although the impact of nucleolin on p53 expression in mouse cells was not confirmed experimentally, our data suggest that the nucleolin–mouse p53 mRNA interaction may be conserved in mice and humans. A majority of the selected proteins (8 out of 15) belong to heterogeneous nuclear ribonucleoproteins (hnRNPs) that can take part in many cellular processes, such as translation regulation, transcription regulation, mRNA stabilization and alternative splicing [60]. Other identified proteins can contribute to transport (RAN [61]), translation (PABP1 [62], FUBP1 [63]), transcription regulation (HMGB1 [64]) or growth regulation (PA2G4 [65]).

### 2.5. hnRNP K and PCBP2 Proteins Efficiently Bind to the 5′-Terminal Region of p53 mRNA

From the group of protein candidates with high likelihood of interacting with mouse p53 mRNA (Table 2) and with the potential to impact the p53 protein synthesis, we selected two proteins for further studies: hnRNP K and PCBP2. Four peptide spectrum matches were assigned to both proteins.

The hnRNP K and PCBP2 proteins are highly conserved. A comparison of hnRNP K protein sequences between mouse and human revealed its 100% identity, and in the case of PCBP2, the sequences differ by only 1.3% (5 out of 365 amino acids).

**Figure 7:**
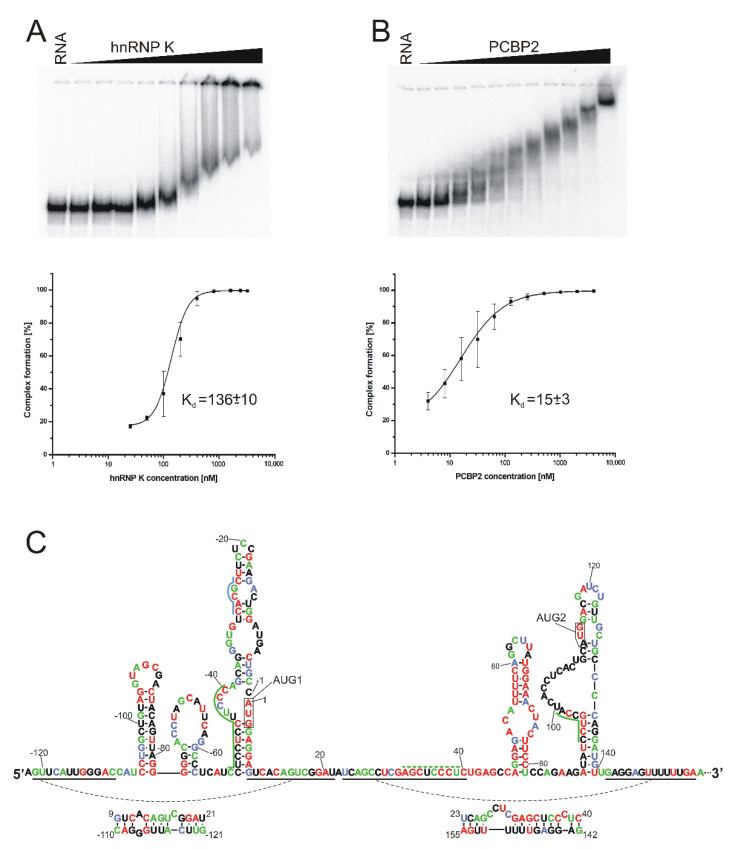
hnRNP K and PCBP2 proteins bind to the 5′-terminal region of p53 mRNA. EMSA assays and graphs representing dissociation constant (K_d_) curves for hnRNP K (**A**) and PCBP2 (**B**). EMSA experiments were conducted by incubation of [^32^P]-labeled RNA-122/201 with increasing concentration of hnRNP K or PCBP2. EMSA products were resolved on 4% polyacrylamide native gels. In the first line of each EMSA panel, RNA was incubated with no protein added. For the calculation of dissociation constants, at least three independent experiments were conducted. (**C**) Secondary structure model of the 5′-terminal region of mouse p53 mRNA with the level of each nucleotide conservation marked based on the alignment of p53 mRNA sequences from eleven different species (modified from [32]) with the proposed binding sites of hnRNP K (blue) and PCBP2 (green) proteins. The nucleotides are colored according to their percentages of conservation (red, 100%; green, 80–99%; blue, 60–79%).

In order to elucidate whether the hnRNP K and PCBP2 proteins are able to directly bind to the 5′-terminal region of mouse p53 mRNA, an electrophoretic mobility shift assay (EMSA) was used. Radiolabeled RNA-122/201 was incubated with hnRNP K or PCBP2 proteins, under three different conditions, and in all cases shifts of the bands were observed on the polyacrylamide gels, which indicated the formation of protein–RNA complexes (data not shown). Afterwards, the EMSA experiments were carried out with an increasing concentration of hnRNP K or PCBP2 in order to estimate the dissociation constant values (K_d_) (Figure 7A,B). A sigmoidal fit model was used in the calculations. For the hnRNP K–RNA-122/201 and PCBP2–RNA-122/201 complexes, the K_d_ values were 136 ± 10 nM and 15 ± 3 nM, respectively. For comparison, the K_d_ value of approximately 50 nM was determined for the binding of hnRNP K to the 5′-terminal region of human p53 mRNA (reference [28]; D. M. Janecki, data not published). The 5′-terminus of human p53 mRNA which starts at the P1 transcription initiation site has two predicted binding sites for the hnRNP K protein: one embedded in the hairpin domain with AUG1 and the other located immediately before AUG2 (28; Figure 7C). Lower binding affinity of hnRNP K to RNA-122/201 can be explained by the lack of the hnRNP K binding site preceding the AUG2 initiation codon in mouse mRNA. A comparison of the degrees of conservation of nucleotide sequences of the 5′-terminus of p53 mRNA in various organisms revealed that mouse and rat mRNA in this region have an additional nucleotide stretch, which disrupts the hnRNP K binding motif present in human mRNA (reference [32]; Figure S6).

The K_d_ value of 95 ± 6 nM reported for the human PCBP2–RNA-P1Δ40p53 complex is higher than K_d_ of 15 ± 3 nM, which was determined for the murine PCBP2–RNA-122/201 complex. It has earlier been described that PCBP2 binding sites in the human 5′-terminal region of p53 mRNA are distributed along this region [29], whereas our analysis of the mouse 5′-terminus of p53 mRNA revealed that all predicted binding sequences are located next to each other, at the bottom-left part of the large hairpin domain with AUG1.

## 3. Discussion

A mouse model has often been used in the studies of p53 gene expression [66,67]. Detailed interpretation of functional studies is, however, hampered by insufficient knowledge of the impact of mouse p53 mRNA’s structure and its interactions with proteins on translation efficiency. In particular, the 5′-terminal region of mouse p53 mRNA is an important region which takes part in the regulation of the synthesis of p53 protein and its N-truncated isoform Δ41p53. A secondary structure model of this mRNA region has recently been proposed, and its important role in translation has been confirmed [32]. In this work, we used the SAXS method and RNAComposer computer program to propose the spatial folding of the 5′-terminal region of mouse p53 mRNA and its selected sub-fragments. Selected fragments of other RNA molecules to study their spatial folds have been employed for SAXS, for example, for studying the HIV-1 5′UTR [68], the apical region of stem-loop IV of poliovirus RNA [69] and hepatitis C virus RNA subdomain 5BSL3.2 [70]. In our studies, special attention was put on the structures of two major functional domains of the 5′-terminal region of mouse p53 mRNA (Figure 1). The AUG1 translation initiation codon was embedded in the first domain, C(-51)/G9, and in the other domain, G47/U140, the Mdm2 binding site and AUG2 initiation codon are present.

The SAXS data show that the C(-51)/G9 domain adopts a bend-shaped structure which corresponds to a large unregular hairpin motif that bends in 2/3 of its length due to the presence of the U(-44):A(-39) bulge (Figure 2). The RNA-38/-2 and RNA-30/-10, which correspond to two upper fragments of this domain, form regular hairpins, and the two symmetric internal bulges do not disturb their double-stranded structures. On the other hand, domain G47/U140 forms a long rod-like structure in which two secondary structure elements, hairpins A47/U81 and A89/U140, are stacked coaxially (Figure 3). Such structure can be modeled by RNAComposer run in the batch mode [35,36]. A coaxial arrangement of two RNA helices located nearby has earlier been observed in other RNAs [68,71]. It is worthy of note that the RNAComposer program run in the interactive mode proposes another structure which is bent in the middle, but this structure does not fit the SAXS-derived ab initio envelope (data not shown). Importantly, the structure generated by RNAComposer for RNA47/81, which forms a regular secondary structure of hairpin type, fits well to the SAXS data. On the other hand, RNA89/140 with AUG2 codon forms a rod-like SAXS-derived ab initio envelope, which is longer than the structure proposed by RNAComposer. Thus, the RNA domain G47/U140 seems to be structurally flexible, and its folding may depend on the surrounding mRNA stretches. Indeed, in the full length 5′-terminus of p53 mRNA, the structure of domain G47/U140 with AUG 2 initiation codon clearly differs from that observed for the corresponding, isolated RNA fragment, for which coaxial stacking of two helical regions is consistent with the SAXS data (Figure 3 and Figure 4). Possibly, the domain is bent to allow interactions with other regions of the mRNA 5′-terminus. On the other hand, the structure of domain C(-51)/G9 in which the AUG1 codon is embedded is similar in both structural environments, as an isolated RNA fragment or as a part of the full length 5′-terminus of mRNA. Importantly, for the full-length 5′-terminal region of p53 mRNA, RNAComposer proposes a spatial structure that fits well to the experimentally generated SAXS-derived ab initio envelope. The envelope is L-shaped, and RNA can be easily accommodated while taking into account the presence of single-stranded flexible stretches in the secondary structure of the 5′-terminal region of p53 mRNA (Figure 4).

Subsequently, we used RNA-assisted affinity chromatography for identification of proteins present in mouse fibroblast NIH3T3 cell lysates that are able to bind the RNA-120/210, which corresponds to the 5′-terminal region of mouse p53 mRNA. In the obtained group of over 80 proteins, there are proteins with the potential to regulate p53 mRNA translation (Table S3). We have recently performed a similar affinity chromatography experiment for the 5′-terminal region of human p53 mRNA and human cell lysates [28]. Table 2 shows if the proteins found in mouse cells were also found in human cell lysates, lines MCF7, HepG2 and HT-29 grown in normal or stress conditions. Importantly, as shown in the table, all the mouse proteins were present in at least three out of six analyzed human samples. Proteins listed in Table S3 include those which have earlier been reported to interact with the 5′-terminus of p53 mRNA, such as hnRNP Q [52], hnRNP L [53], ribosomal protein RPL26 [54] and very recently also hnRNP K [28] and PCBP2 [29]. All the proteins listed in Table 2 can potentially regulate *p53* gene expression by influencing the p53 translation initiation process. Moreover, our results suggest a general homology of proteins that bind to the 5′-terminus of p53 mRNA between mice and humans. The results also confirm the effectiveness of RNA-centric affinity chromatography combined with an MS approach in catching out the protein candidates which potentially can influence translation process.

Possible sites in the 5′-terminal region of mouse p53 mRNA to which the 15 identified proteins bind are shown in Figure 6. They are located mostly in loops and single-stranded regions or regions of lower stability due to the presence of non-standard base interactions or adjacent single-stranded RNA stretches. Interestingly, most of these binding sites coincide with the sites determined as accessible to hybridization of complementary oligonucleotides (Figure 5 and Figure S3). In particular, this applies to the apical loop regions of hairpins C(-106)/G(-78) and A89/U140; and the bulges and double-stranded stretches of bottom-left segments of hairpins U(-50)/A8 and A89/U140. More than one protein from the list shown in Table 2 is bound to each of these accessible regions. The sites accessible to hybridization of complementary oligonucleotides were determined with semi-random DNA libraries and RNase H approach [40,41]. This approach has been shown especially useful in screening for target sites for antisense oligonucleotides in highly structured RNA molecules, such as genomic and antigenomic HDV ribozyme [40] or region X of hepatitis C virus [51]. Moreover, semi-random DNA libraries and RNase H have also been used for mapping the accessibility of a region homologous to our model, namely, the 5′-terminal region of human p53 mRNA, which commences at the P1 transcription initiation site [20]. Earlier published data and the results of this work show that semi-random DNA libraries and RNase H approach are very useful in the identification of highly accessible sites in RNA and sites prone to bind trans-acting factors, such as antisense oligonucleotides or proteins.

Finally, we focused on two proteins identified in the RNA-assisted affinity chromatography experiment that are members of the poly(C)-binding proteins family (PCBP), hnRNP K and PCBP2 proteins. As has already been described, these proteins can participate in several posttranscriptional regulation processes, such as translational silencing, translational enhancement or mRNA stabilization [72]. A characteristic feature of proteins belonging to the PCBP family is the presence of three KH domains of about 70 amino acids each. Two out of the three domains, KH1 and KH3, can interact with cytosine tracks in RNA in a sequence-specific manner [72,73,74]. Human hnRNP K and PCBP2 proteins have recently been identified in our laboratory as interacting with the 5′-terminal region of human p53 mRNA, and their impact on mRNA translation has been confirmed [28,29]. Consequently, an interaction of human hnRNP K and PCBP2 with the 5′-terminus of murine p53 mRNA provides evidence for the analogy to what we can observe in human cells. Indeed, we determined high binding affinity of hnRNP K and PCBP2 to the 5′-terminal region of mouse p53 mRNA using the EMSA approach (Figure 7A,B). The corresponding dissociation constant K_d_ for hnRNP K was 136 nM, and for PCBP2 K_d_, it was 15 nM.

There is only a single binding site for hnRNP K predicted in the 5′-terminal region of mouse p53 mRNA (Figure 7C). This site is located between C(-29) and C(-25) in the C(-51)/G9 domain. Out of the five nucleotides of this binding site, four nucleotides are perfectly conserved (Figure S6). The nucleotides that correspond to those present in mouse p53 mRNA have also been proposed as a hnRNP K binding place in human p53 mRNA [28]. Another binding site has also been proposed to be in this mRNA in the region that proceeds the AUG2 initiation codon (Figure S6). This site is, however, absent in the corresponding region of mouse mRNA, which forms a large bulge in this region of the mRNA secondary structure. The bulge nucleotides are little conserved. Thus, in both mouse and human p53 mRNAs, hnRNP K seems to bind to the upper part of domain C(-51)/G9 in which AUG1 is embedded. An additional binding site close to AUG2 is proposed only in human mRNA. It needs to be further determined whether there is another hnRNP K binding site in mouse p53 mRNA.

Two binding sites for PCBP2 are proposed to be in the 5′-terminal region of mouse p53 mRNA (Figure 7C). The first site is located on the 5′ side of the bottom part of domain C(-51)/G9. No binding site for PCBP2 has been proposed for the corresponding region of human mRNA. This might be a consequence of an additional 3-nucleotide tract UCC which is present only in mouse mRNA out of eleven other p53 mRNAs from various organisms (Figure S6). The other PCBP2 binding site in mouse p53 mRNA is present at the junction of the single- and double-stranded region at the large bulge in domain A89/U140. Most nucleotides of this binding site are perfectly conserved (Figure 7C). In human p53 mRNA, this site seems to be preserved, despite some differences in nucleotide sequence. Moreover, there is another PCBP2 binding site in the upstream region of human mRNA which is not present in mouse p53 mRNA. Importantly, an additional PCBP2 binding site has been proposed to be in human mRNA which corresponds to the nucleotide stretch between A32 and U39 in mouse mRNA. Nucleotides of this region are almost perfectly conserved, and only the middle C residue is replaced by U35 in mouse mRNA. Taking into account this single nucleotide substitution and the very high conservation of this region, one might predict the presence of a PCBP2 binding site also in mouse p53 mRNA.

Our findings also have some important consequences for the studies of interactions between other proteins and the 5′-terminal region of p53 mRNA. Some binding sites for a given protein in human and mouse mRNAs (in our studies for hnRNR K or PCBP2) closely correspond to each other; however, some other sites occur only either in human or mouse mRNA. This has to be taken into consideration while planning RNA mutagenesis experiments designed to decipher specific protein–RNA interactions, especially for proteins with multiple RNA-binding domains.

## 4. Materials and Methods

### 4.1. DNA Template Constructs and RNA Synthesis

DNA templates for RNA synthesis were prepared via PCR reaction using primers, whose sequences are summarized in Table S1. Each forward primer contained part of corresponding RNA sequence followed by the T7 transcription promoter sequence. Synthesis of the templates for RNA-51/9 and RNA89/140 was described earlier [32]. Before the PCR reaction, all primers were purified on 8% polyacrylamide gels. For the synthesis of RNA-122/201 and full-length p53 mRNA, a plasmid containing a mouse p53 nucleotide sequence was digested using restriction enzyme Psp5II or NotI (Thermo Scientific, Waltham, MA, USA), respectively. Sequences spanned the 5′UTR (122 nucleotides in length), and a coding sequence and the 3′UTR. Preparation of the plasmid has been described in our previous work [32].

In vitro transcription was conducted in order to synthetize RNA using a TranscriptAid T7 High Yield Transcription Kit (Thermo Scientific), following the manufacturer’s protocol. After transcription reactions, RNA was incubated with DNase I at 37 °C for 20 min and subsequently cleaned using a GeneJET RNA Cleanup and Concentration Micro Kit (Thermo Scientific). The RNA integrity and size were assessed using agarose gel electrophoresis.

Prior to the SAXS experiment and CD measurements, RNA was suspended in water at 1.7 mg/mL (16.3 µM for RNA-122/201) to 4.1 mg/mL (232 µM for RNA47/140). The sample was heated at 95 °C for 1 min, and then incubated for 5 min at room temperature before adding an equal amount of 2-fold concentrated folding buffer to the RNA. Finally, samples were incubated for 10 min at room temperature and measured. The folding buffer consisted of 40 mM Tris at pH 7.5, 130 mM KCl, 0.5 mM EDTA and MgCl_2_ at 0, 2.5 or 5 mM.

### 4.2. Mapping of RNA Accessibility to Hybridization with DNA 6-Mer Libraries and RNase H Cleavage

Mapping p53 RNA accessibility to oligomer hybridization was performed as described earlier [20,40,41]. Briefly, before the digestion with RNase H (Thermo Scientific), 0.5 pmol of full-length p53 mRNA was incubated in the buffer—20 mM Tris-HCl pH 7.8, 40 mM KCl, 8 mM MgCl_2_ and 1 mM DTT at 65 °C—for 2 min and then cooled to 37 °C. Subsequently, RNase H was added to the final concentration of 225 units/mL. The cleavage reactions were induced by separately adding four DNA 6-mer libraries (final concentration of 100 µM) to four RNA samples. All reaction mixtures were incubated at 37 °C for 10 or 30 min. The reactions were stopped via RNase H inactivation by heating at 65 °C for 10 min. Subsequently, the reaction products were purified by phenol/chloroform (1:1) extraction and precipitated overnight with 3 volumes of ethanol, 0.3 M sodium acetate at pH 5.2 and 1 µL of glycogen (20 mg/mL).

In order to visualize RNase H cleavages, primer extension analysis was carried out using the following 5′-end-[^32^P]-labeled primers: Rm508 (5′-TTCGGAGAAGCGTGACACCCTGC-3′), Rm591 (5′-CCATAAGCCTGAAAATGTCTCCT-3′) and Rm679 (5′-TTCAAAAAACT CCTCAACATCCT-3′) and 50 units of Super Script IV reverse transcriptase (Invitrogen, Waltham, MA, USA). The reverse transcription products were separated on 8% polyacrylamide gels and visualized with the FLA 5100 image analyzer (Fuji, Tokyo, Japan).

### 4.3. Preparation of Cytoplasmic Lysate

Cytoplasmic lysate was prepared as described earlier [28]. For a single RNA-affinity chromatography experiment, about 4 × 10^7^ NIH3T3 cells were used. At first, all cells were washed with PBS buffer and then harvested and collected by centrifugation at 1000 rpm. In the next step, the pellet was resuspended in CE buffer: 10 mM HEPES, 60 mM KCl, 1 mM EDTA, 0.075% (*v*/*v*) NP40, 1 mM DTT and 1 mM PMSF, pH 7.6, followed by a 5 min incubation on ice. Finally, the prepared extract was centrifuged at 1200 rpm for 5 min, and cytoplasmic extract was collected.

### 4.4. RNA-Centric Affinity Chromatography

RNase-assisted RNA chromatography was performed according to the protocols described earlier [28,37]. Briefly, 1.35 nM RNA-122/201 was incubated in a reaction medium (200 µL) containing 0.1 M NaOAc pH 5.0 and 5 mM sodium m-periodate (Sigma, St. Louis, MO, USA) at room temperature, in the dark, for 1 h. Subsequently, RNA was ethanol precipitated and resuspended in 0.1 M NaOAc pH 5.0, followed by addition of an adipic acid, dehydrazide agarose beads slurry suspended in 0.1 M NaOAc pH 5.0.

The prepared RNA was incubated with the beads overnight at 4 °C in order to conjugate RNA with the beads. The obtained complex was washed three times in 2 M KCl and three times with buffer D: 20 mM Tris-HCl pH 7.9, 20% (p/v) glycerol, 0.1 M KCl, 0.2 mM EDTA, 0.5 mM dithiothreitol, 0.2 mM PMSF. The RNA conjugated with the beads was incubated with 40% (*v/v*) NIH3T3 cytoplasmic extract with 1.5 mM MgCl_2_ and 25 mM creatine-phosphate, and 5 mM ATP was added. Incubation was conducted for 30 min at 37 °C with shaking at 400 rpm. Subsequently, the beads were washed four times with buffer D containing 1.5 mM MgCl_2_ and twice with Mili-Q water. Next, the RNA combined with the beads was incubated in the reaction medium (60 µL total volume) which contained 10 mM Tris-HCl pH 7.2, 1 mM MgCl_2_, 40 mM NaCl and 5 μL of A/T1 ribonuclease mix (Ambion, Austin, TX, USA, 500 U/mL of RNase A, 20,000 U/mL of RNase T1). The reaction sample was incubated at 37 °C for 30 min, with shaking at 1400 rpm for 10 sec every 10 min. Finally, the sample was centrifuged for 1 min at 4 °C, and supernatant was collected.

### 4.5. Mass Spectrometry Analysis

After RNase-assisted RNA chromatography, protein identification was conducted using MS/MS analysis. The analysis was carried out in a mass spectrometry laboratory (IBB PAS, Warsaw, Poland). All the results were uploaded to the Mascot program (Matrix Science, London, UK), and searching was performed in the SwissProt *Mus musculus* database. Next, the list of the obtained results was analyzed using MScan software (http://proteom.ibb.waw.pl/mscan (accessed on 25 May 2022). Only proteins with at least two peptides with a score above the threshold were analyzed. The equipment used was sponsored in part by the Centre for Preclinical Research and Technology (CePT), a project co-sponsored by the European Regional Development Fund and the Innovative Economy Programme, The National Cohesion Strategy of Poland.

### 4.6. CD Measurements

Circular dichroism spectra were collected on a J-815 CD spectrometer (JASCO, Tokyo, Japan) equipped with a Peltier thermostatic cell holder. The RNA solution in a buffer containing 20 mM Tris pH 7.5, 65 mM KCl, 0/5 mM MgCl_2_ and 0.25 mM EDTA was analyzed in a 0.2 cm quartz cuvette (Hellma 100-QS, Jena, Germany). Each CD spectrum was generated based on nine scans in a continuous scanning mode, with the scanning speed of 50 nm min^−1^, a 1 nm bandwidth, a 0.5 nm data pitch and a data integration time of 1 sec. Data were collected at wavelengths ranging from 200 to 350 nm when collecting a regular spectrum, or at wavelengths ranging from 200 to 300 nm for the thermal melt analysis. Thermal analysis was performed in the temperature range of 5 to 90 °C. Different buffers were used for different conditions. Buffer subtraction and all spectrum processing were performed using the Jasco Spectra Manager software using Savitzky-Golay tool with a smoothing window of 10 points. The normalized root mean square deviation (NRMSD) for each CD spectrum analysis was less than 0.1. CD data are presented in terms of ellipticity in millidegrees (mdeg) or as the mean residue ellipticity in deg cm^2^ dmol^−1^.

### 4.7. Small Angle X-ray Scattering

Small angle X-ray scattering pattern curves were collected at beamline P12 of the Petra III storage ring at the DESY (Deutsches Electronen Synchrotron, Hamburg, Germany) in Hamburg, Germany. Twenty-microliter samples of RNA solution and of the corresponding matching buffer (20 mM Tris pH 7.5, 65 mM KCl, 0/2.5/5 mM MgCl_2_, 0.25 mM EDTA) were analyzed. All data were collected at 15 °C. SAXS data were collected over the s range of 0.0088–5 nm^−1^. Experimental SAXS data were processed using the ATSAS package [75]. The PRIMUS program was used for the integration, scaling and buffer subtraction. The DAMMIF program was used for ab initio modeling [76]. Automated matching of high and low resolution structural models was performed using the SUPCOMB program [77]. All 3D visualizations were prepared using the PyMOL software v0.99 (DeLano Scientific LLC, South San Francisco, CA, USA).

### 4.8. Generation of RNA Fragments’ Tertiary Structure Models

High resolution tertiary structure models of RNAs: -106/-78, -30/-10, -38/-2, -51/9, 47/81, 47/140, 89/140 and -122/201 were generated using the RNAComposer modeling server (http://rnacomposer.cs.put.poznan.pl/ (accessed on 19 May 2022) [35,36]. In order to obtain appropriate RNA structure models, nucleotide sequence and secondary structure in dot-bracket notation were uploaded. In the case of RNA-122/201, long-range interactions were also marked.

### 4.9. Protein Overexpression and Purification

hnRNP K and PCBP2 proteins were overexpressed and purified earlier in our laboratory, according to the protocol previously described [28,29].

### 4.10. Electrophoretic Mobility Shift Assay

Before protein–RNA complex formation, RNA-122/201 was radiolabeled with γ-[^32^P] ATP at the 5′ end using T4 polynucleotide kinase, following the standard procedure. Then, the RNA was denatured at 65 °C for 5 min and cooled on ice. The RNA–protein binding reaction was conducted in a buffer containing 25 mM NaH_2_PO_4_ pH 8.0, 50 mM NaCl, 25 mM KCl, 2.5 mM MgCl_2_, 10 mM imidazol, 0.5 mM DTT, 0.5 mM PMSF, 7% glycerol, 0.5 U/μL RNasin, 75 ng/μL BSA and 100 ng/μL yeast tRNA for PCBP2 protein; and 150 mM NaCl, 25 mM KCl, 50 mM Tris-HCl pH 8.0, 5 mM HEPES-KOH pH 8.0, 10 mM imidazole, 5 mM MgCl_2_, 0.5 mM DTT, 0.5 mM PMSF, 75 ng/µL BSA, 100 ng/µL yeast tRNA, 7% glycerol and 0.5 U/μL RNasin for hnRNP K protein. In order to measure the K_d_ values, [^32^P]-labeled RNA-122/201 was incubated for 25 min at 4 °C with the protein at the following concentrations: 3.9, 7.8, 15.6, 31.3, 62.5, 125, 250, 500, 1000, 2000 and 4000 nM for PCBP2; and 25, 50, 100, 200, 400, 800, 1600, 2400 and 3200 nM for hnRNP K. Samples were loaded on 4% polyacrylamide gels (acrylamide to bisacrylamide 29:1, 2,5% glycerol and TB buffer). Electrophoresis was conducted for 3 h at 4 °C, the gel was dried and the results were visualized using FLA 5100 image analyser (Fuji).

## Figures and Tables

**Table 1:** SAXS data collection and structural parameters for RNA -30/-10, -38/-2, -51/9, 47/81, 89/140, 47/140 and -122/201.

**Sample**	-30/-10	-38/-2	-51/9	47/81	89/140	47/140	-122/201
**Data collection**							
Instrument	P12, PETRA III						
s range (nm^−1^)	0.0088–5.0						
Wavelength (Å)	1.24						
Temperature	15 °C						
**Structural parameters**							
R_g_ (from p(r)) (nm)	1.5 ± 0.05	1.94 ± 0.02	4.10 ± 0.02	2.078 ± 0.1	4.34 ± 0.02	6.17 ± 0.05	9.84 ± 0.04
R_g_ (from Guinier) (nm)	1.46 ± 0.03	1.96 ± 0.23	3.87 ± 0.44	2.099 ± 0.1	4.01 ± 0.31	6.02 ± 0.134	9.78 ± 0.34
R_g_ (theoretical) (nm)	1.275	1.744	2.484	1.73	1.985	5.498	9.535
D_max_ (nm)	5.07	6.32	16.21	7.1	17.65	26.88	30.00
D_max_ (theoretical)	4.169	6.145	8.006	6.404	6.674	19.87	34.84
Porod volume estimate (nm^3^)	9697	14,286	21,908	11,593	20,641	38,167	126,618
Dry volume calculated from model (nm^3^)	8081	11,905	19,051	9661	16,782	31,806	101,295
**Molecular mass determination**							
Contrast (Δρ × 10^10^ cm^−2^)	3.047						
Experimental molecular weight (Da)	7125	12,759	20,187	11,238	17,342	32,014	10,128
Theoretical molecular weight (Da)	6351	11,623.4	18,575.7	10,919.9	16,289.3	30,997.6	98,792.2
**Software used**							
Primary data reduction	PRIMUS						
Data processing	PRIMUS						
Quaternary structure modelling	SASREF						
Computation od model intensities	CRYSOL						
3D graphics representation	PyMOL						

**Table 2:** List of selected proteins interacting with the 5′-terminus of p53 mRNA (this work) compared to proteins present in human cell lines MCF7, HepG2 and HT-29, as identified in [28].

No.	ID	Protein	Aliases	MS Spectra Number	Cell Line	MCF7		HepG2		HT-29	
					Doxo	-	+	-	+	-	+
1	P09405	Nucleolin	-	40		+	+	+	+	+	+
2	P29341	Polyadenylate-binding protein 1	PABP1	24		+	+	-	+	-	-
3	P62827	GTP-binding nuclear protein Ran	RAN	22		-	+	+	+	+	+
4	P49312	Heterogeneous nuclear ribonucleoprotein A1	hnRNP A1	20		+	+	+	+	+	+
5	O88569	Heterogeneous nuclear ribonucleoproteins A2/B1	hnRNP A2/B1	18		+	-	+	+	+	+
6	P50580	Proliferation-associated protein 2G4	PA2G4, EBP1	17		+	+	-	-	+	+
7	Q60668	Heterogeneous nuclear ribonucleoprotein D0	hnRNP D0	13		+	+	+	+	+	+
8	P97855	Ras GTPase-activating protein-binding protein 1	G3BP1	12		+	+	-	-	+	-
9	P63158	High mobility group protein B1	HMGB1	9		+	+	-	+	+	+
10	Q8BG05	Heterogeneous nuclear ribonucleoprotein A3	hnRNP A3	8		+	-	+	+	-	+
11	Q91WJ8	Far upstream element-binding protein 1	FUBP1, FUP	7		-	-	+	+	+	+
12	P17225	Polypyrimidine tract-binding protein 1	PTBP1	7		+	+	+	+	+	+
13	Q61990	Poly(rC)-binding protein 2	PCBP2	4		+	+	-	-	+	+
14	P61979	Heterogeneous nuclear ribonucleoprotein K	hnRNP K	4		+	+	+	+	+	+
15	Q8BHD7	Polypyrimidine tract-binding protein 3	PTBP3, ROD1	2		-	+	+	+	+	+

## Data Availability

The data sets and materials generated during and/or analyzed during the current study are available from the corresponding author on reasonable request.

## Supplementary Materials

The following are available online at www.mdpi.com/article/10.3390/ijms23179709/s1.
