# Supplementary material for: Structural Characteristics of the 5′-Terminal Region of Mouse p53 mRNA and Identification of Proteins That Bind to This mRNA Region
Source: Int J Mol Sci. 2022 Aug 26;23(17):9709. doi: 10.3390/ijms23179709 (PMC9456389; doi:10.3390/ijms23179709)
Supplement: Supplementary file 1 [file ijms-23-09709-s001.zip › ijms-1807552-supplementary.pdf]

Supplementary materials

Structural characteristics of the 5'-terminal region of mouse p53 mRNA and identification of proteins that bind to this mRNA region

Joanna Szpotkowska [1,2,†], Kamil Szpotkowski [1,†], and Jerzy Ciesiołka [1]*

[1]Institute of Bioorganic Chemistry, Polish Academy of Sciences, 61-704 Poznan, Poland
[2]Department of Gene Expression, Faculty of Biology, Institute of Molecular Biology and Biotechnology, Adam Mickiewicz University Poznan, 61-614, Poznan, Poland

***** Correspondence: jerzy.ciesiolka@ibch.poznan.pl

†These authors contributed equally to this work.

Table S1. List of primers used to synthetize RNAs: -30/-10, -38/-2, 47/81, and 47/140. The T7 transcription promoter sequence is marked in italics.

Name	Sequence (5'-3')
-30/-10F	*TAATACGACTCACTATAGG*CACGCTTCTCC
-30/-10R	CAGTCTTCGGAGAAGCGTGCCTATAGTGAGTC
-38/-2F	*TAATACGACTCACTATAG*GCAGGGTGTCACGCTTCTCCGAAG
-38/-2R	GCAGTCATCCAGTCTTCGGAGAAGCGTGACACCCTGC
47/140F	*TAATACGACTCACTATAGG*TGAGCC*AGGAGACATTTTCAGGCTTA*
47/140R	ACATCCTGGGGCAGCAACAGAT
47/81F	*TAATACGACTCACTATAG*AGGAGACATTTTCAGGCTT
47/81R	AGGAAGTAGTTTCCATAAGCCTGAAAATGTCTCCT

Table S2. Structural parameters derived from SAXS data for RNA-106/-78 and RNA-30/-10 in three Mg^{2+} concentrations.

RNA	Mg^{2+} concentration [nM]	R_g (from p(r)) (nm)	R_g (from Guinier) (nm)	D_{max} (nm)
-106/-78	0	1.72±0.06	1.84±0.28	5.17
	2.5	2.08±0.06	2.04±0.28	7.10
	5	1.97±0.06	1.97±0.23	6.45
-30/-10	0	1.32±0.03	1.36±0.12	4.31
	2.5	1.49±0.03	1.52±0.11	4.64
	5	1.50±0.05	1.46±0.03	5.07

Abbreviations: R_g – Radius of gyration, D_{max} – maximum dimension

Table S3. List of candidate proteins identified by RNA-assisted chromatography combined with MS analysis.

No.	ID	Name	sample	control
1	P09405	Nucleolin	40	-
2	P29341	Polyadenylate-binding protein 1	24	7
3	P62827	GTP-binding nuclear protein Ran	22	-
4	P49312	Heterogeneous nuclear ribonucleoprotein A1	20	-
5	O88569	Heterogeneous nuclear ribonucleoproteins A2/B1	18	-
6	P50580	Proliferation-associated protein 2G4	17	-
7	P47962	60S ribosomal protein L5	16	-
8	Q9CY58	Plasminogen activator inhibitor 1 RNA-binding protein	16	1
9	Q99020	Heterogeneous nuclear ribonucleoprotein A/B	14	-
10	Q60668	Heterogeneous nuclear ribonucleoprotein D0	13	-
11	P97855	Ras GTPase-activating protein-binding protein 1	12	-
12	Q9JKB3	Y-box-binding protein 3	12	-
13	Q64152	Transcription factor BTF3	10	-
14	P63158	High mobility group protein B1	9	1
15	Q80X50	Ubiquitin-associated protein 2-like	9	2
16	P28352	DNA-(apurinic or apyrimidinic site) endonuclease	9	-
17	P70670	Nascent polypeptide-associated complex subunit alpha, muscle-specific form	8	-
18	Q60817	Nascent polypeptide-associated complex subunit alpha	8	-
19	Q8BG05	Heterogeneous nuclear ribonucleoprotein A3	8	-
20	Q91VU7	Pseudouridylate synthase 7 homolog	7	-
21	Q61820	GTP-binding nuclear protein Ran, testis-specific isoform	7	-
22	Q91WJ8	Far upstream element-binding protein 1	7	-
23	P62960	Y-box-binding protein 1	7	-
24	P17225	Polypyrimidine tract-binding protein 1	7	-
25	Q8CHP5	Partner of Y14 and mago	7	-
26	Q8VDJ3	Vigilin	7	-
27	O89086	RNA-binding protein 3	6	-
28	Q5SF07	Insulin-like growth factor 2 mRNA-binding protein 2	6	-
29	Q91W59	RNA-binding motif, single-stranded-interacting protein 1	6	-
30	P84104	Serine/arginine-rich splicing factor 3	6	-
31	P56959	RNA-binding protein FUS	5	-
32	O08663	Methionine aminopeptidase 2	5	-
33	Q61545	RNA-binding protein EWS	5	-
34	Q6PDM2	Serine/arginine-rich splicing factor 1	5	-
35	P51859	Hepatoma-derived growth factor	5	-
36	Q8VC70	RNA-binding motif, single-stranded-interacting protein 2	5	-
37	Q9Z2X1	Heterogeneous nuclear ribonucleoprotein F	5	-
38	Q99JF8	PC4 and SFRS1-interacting protein	5	-
39	P30681	High mobility group protein B2	4	-
40	Q3U0V1	Far upstream element-binding protein 2	4	-
41	Q61990	Poly(rC)-binding protein 2	4	-
42	P61979	Heterogeneous nuclear ribonucleoprotein K	4	-
43	P20152	Vimentin	4	-

44	Q6P9R2	Serine/threonine-protein kinase OSR1	4	-
45	Q9Z1W9	STE20/SPS1-related proline-alanine-rich protein kinase	4	-
46	Q9Z130	Heterogeneous nuclear ribonucleoprotein D-like	4	-
47	Q9CPN8	Insulin-like growth factor 2 mRNA-binding protein 3	3	-
48	P70372	ELAV-like protein 1	3	-
49	Q9D0E1	Heterogeneous nuclear ribonucleoprotein M	3	-
50	Q60899	ELAV-like protein 2	3	-
51	O35737	Heterogeneous nuclear ribonucleoprotein H	3	-
52	Q99LF4	RNA-splicing ligase RtcB homolog	3	-
53	Q61937	Nucleophosmin	3	-
54	P17095	High mobility group protein HMG-I/HMG-Y	3	-
55	Q9JMG7	Hepatoma-derived growth factor-related protein 3	3	-
56	P97379	Ras GTPase-activating protein-binding protein 2	3	-
57	Q08093	Calponin-2	3	-
58	P16858	Glyceraldehyde-3-phosphate dehydrogenase	3	-
59	P63013	Paired mesoderm homeobox protein 1	2	-
60	Q9Z2C8	Y-box-binding protein 2	2	-
61	P57722	Poly(rC)-binding protein 3	2	-
62	O54879	High mobility group protein B3	2	-
63	P84078	ADP-ribosylation factor 1	2	-
64	P61205	ADP-ribosylation factor 3	2	-
65	Q60865	Caprin-1	2	-
66	P24369	Peptidyl-prolyl cis-trans isomerase B	2	-
67	Q8QZY9	Splicing factor 3B subunit 4	2	-
68	O70310	Glycylpeptide N-tetradecanoyltransferase 1	2	-
69	P07356	Annexin A2	2	-
70	O35295	Transcriptional activator protein Pur-beta	2	-
71	P63101	14-3-3 protein zeta/delta	2	-
72	P60335	Poly(rC)-binding protein 1	2	-
73	Q5SW19	Clustered mitochondria protein homolog	2	-
74	Q501J6	Probable ATP-dependent RNA helicase DDX17	2	-
75	Q8BHD7	Polypyrimidine tract-binding protein 3	2	-
76	Q8BWL5	RNA-binding motif, single-stranded-interacting protein 3	2	-
77	Q9JII5	DAZ-associated protein 1	2	-
78	Q9CQH7	Transcription factor BTF3 homolog 4	2	-
79	Q923D5	WW domain-binding protein 11	2	-
80	P07901	Heat shock protein HSP 90-alpha	2	-
81	P11499	Heat shock protein HSP 90-beta	2	-
82	P0C5E4	Phosphatidylinositol phosphatase PTPRQ	2	-
83	Q0VFX2	Cilia- and flagella-associated protein 157	2	-
84	Q3U3T8	WD repeat-containing protein 62	2	-

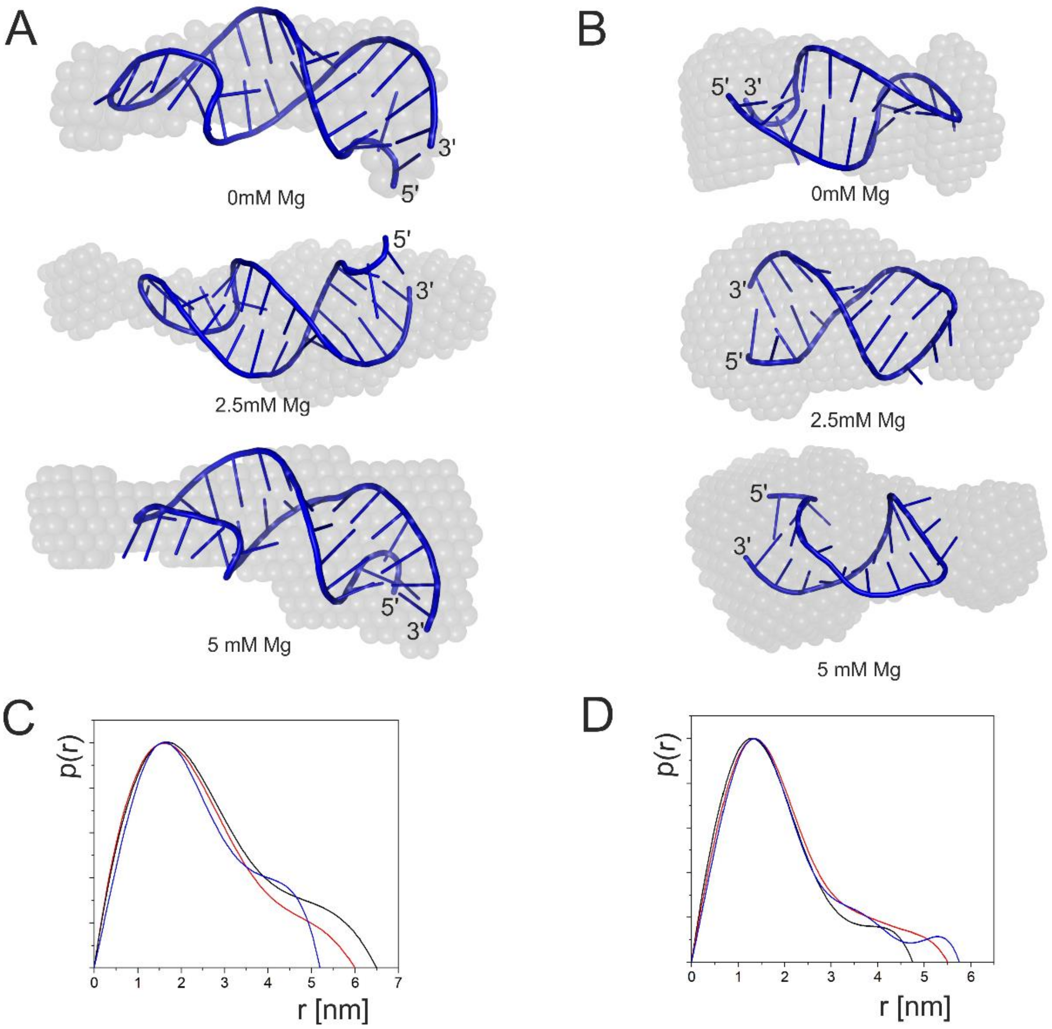

Figure S1. The impact of magnesium ions on mRNA fragments structure. Evolution of *ab initio* structure of RNA-106/-78 **(A)** and RNA-30/-10 **(B)** upon increasing concentration of magnesium ions. Changes of the shape of the pair distance distribution function corresponding to the *ab initio* shape of RNA-106/-78 **(C)** and RNA-30/-10 **(D)**: black – 0 mM Mg^{2+}, red – 2,5 mM Mg^{2+}, blue – 5 mM Mg^{2+}.

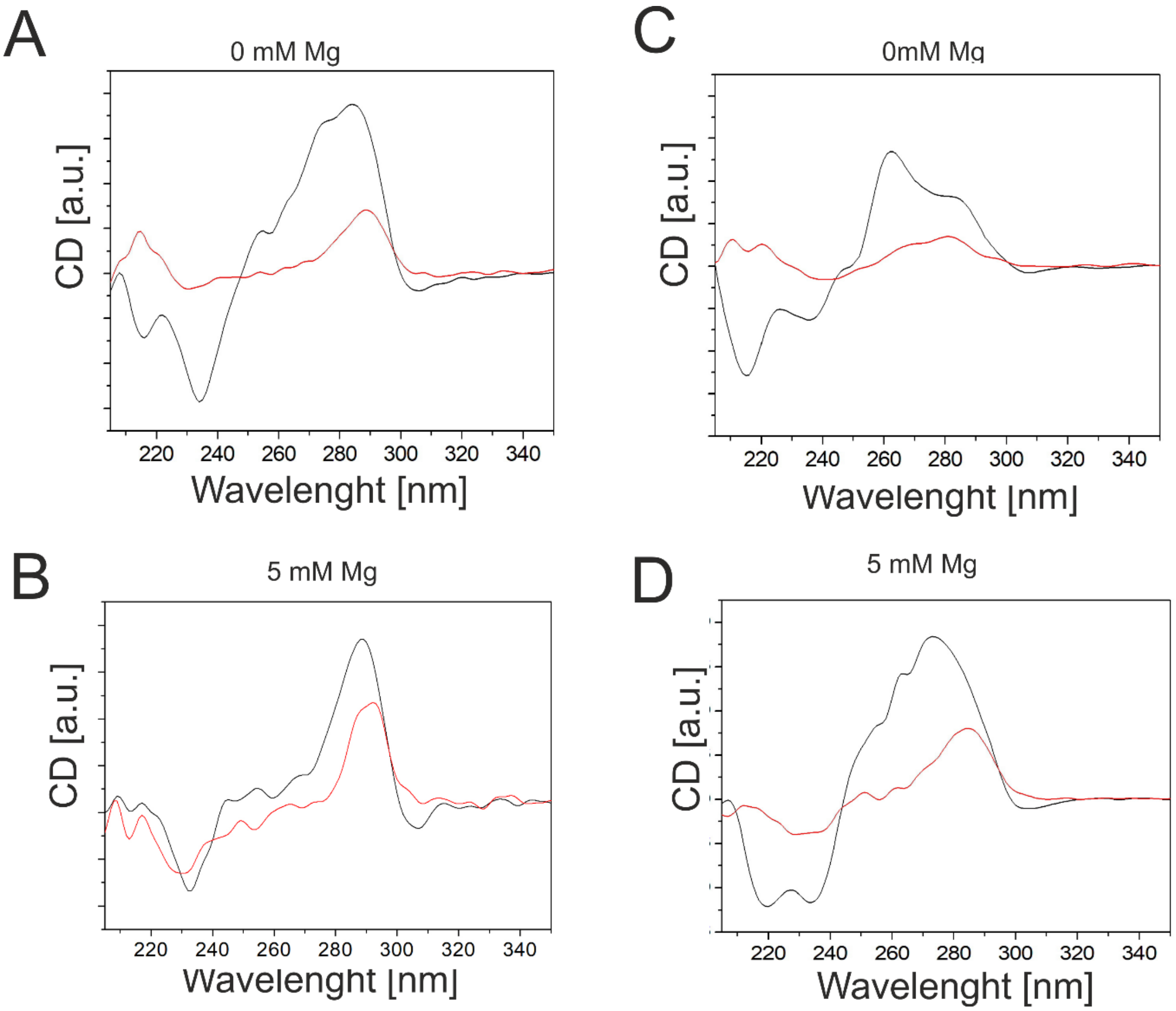

Figure S2. Circular dichroism spectra of RNA -30/-10 (A and B) RNA -106/-78 (C and D) in a buffer with and without Mg^{+2} ions at two different temperatures: 25°C and 85°C (black and red curves, respectively). All spectra have a broad positive maximum covering the region from 260 to 300 nm and negative peaks at 210 and 240 nm.

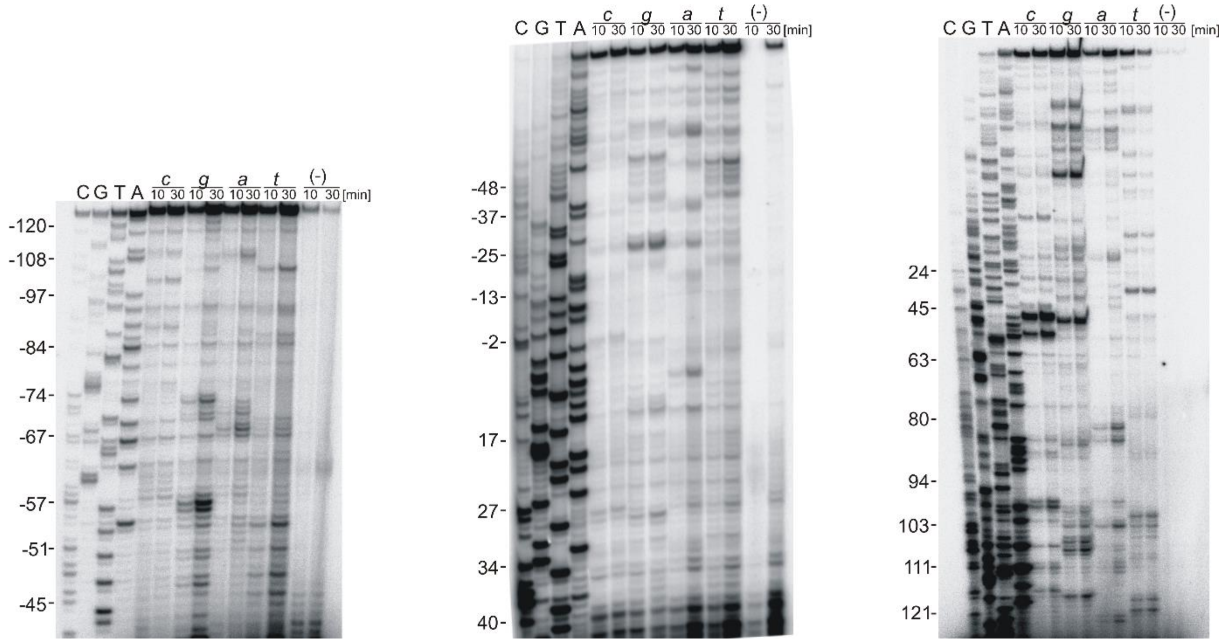

Figure S3. RNA accessibility mapping to oligonucleotide hybridization. Autoradiograms show RNase H cleavage sites identified by reverse transcription with 5'end-[^{32}P]-labelled DNA primers. Selected cytosine and adenosine residues are presented on the left side of each autoradiogram. Sequencing lines are marked as C, G, T, A. *c*, *g*, *t*, *a* – semi-random libraries with one nucleotide residue fixed in the third position. Incubation time for each reaction is also shown.

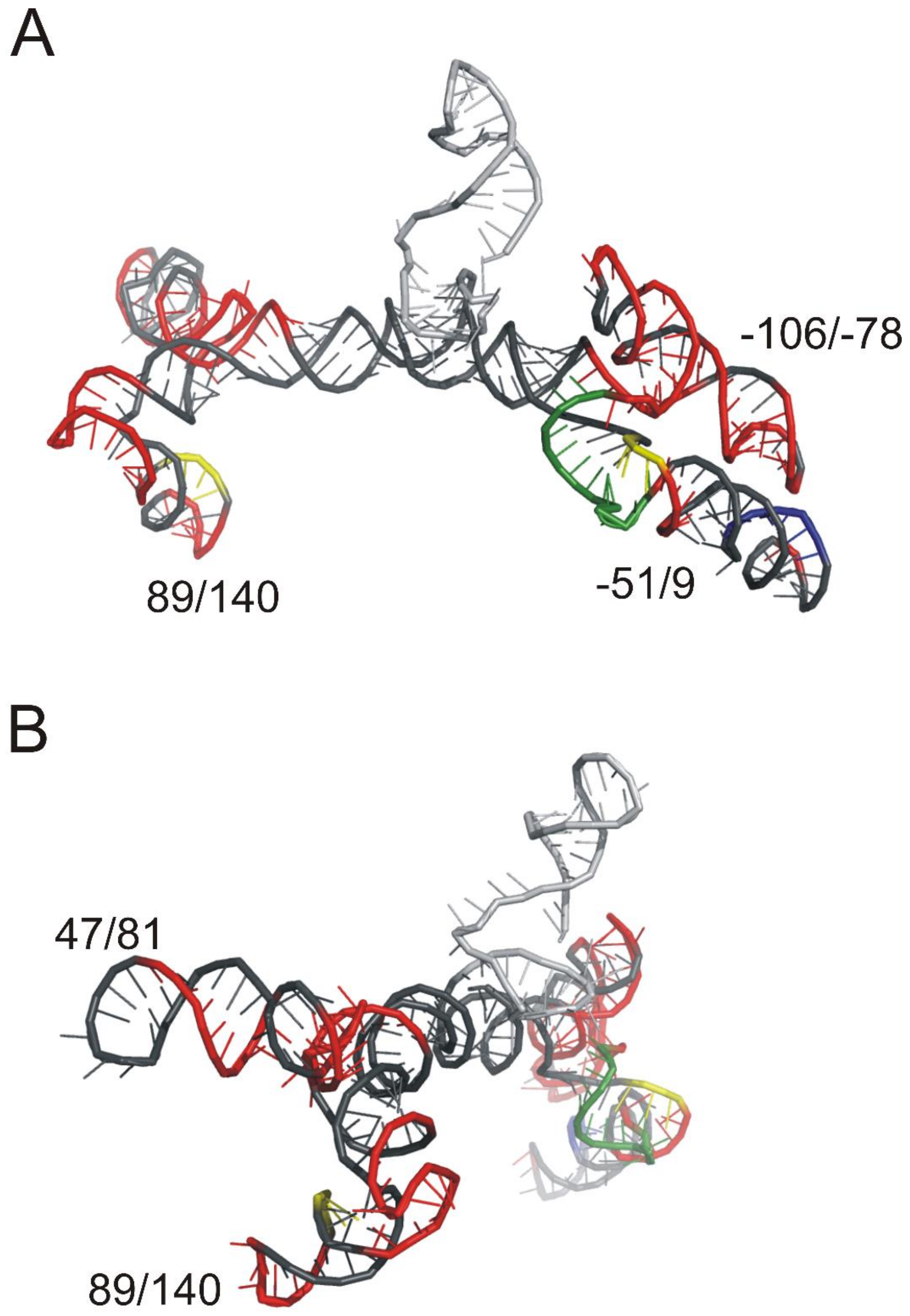

Figure S4. Mapping of RNA accessibility to hybridization of complementary oligonucleotides. Short semi-random DNA libraries and RNase H cleavage approach were used to map sites accessible to hybridization in the 5’-terminal region of mouse p53 mRNA. Only nucleotides where at least three oligomers potentially bind to RNA are shown in the figure in red color. AUG1 and AUG2 codons are marked in yellow, binding sites for hnRNP K and PCBP2 proteins are indicated in blue and green, respectively. Accessibility of mRNA regions C(-106):G(-78) and C(-51):G9 **(A)** and G47:U81 and G89:U140 **(B)** is visualized.

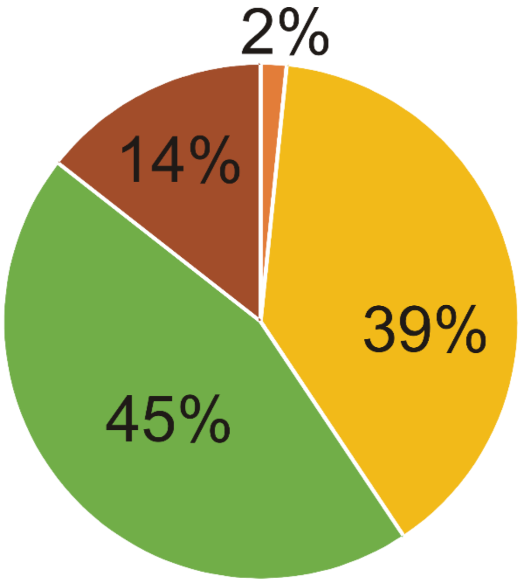

Figure S5. Pie chart representing distribution of proteins identified by RNA-assisted affinity chromatography combined with mass spectrometry for NIH3T3 cells. Candidates – 45% (green), translation machinery – 39% (yellow), histones – 14% (brown), known interactions – 2% (orange).

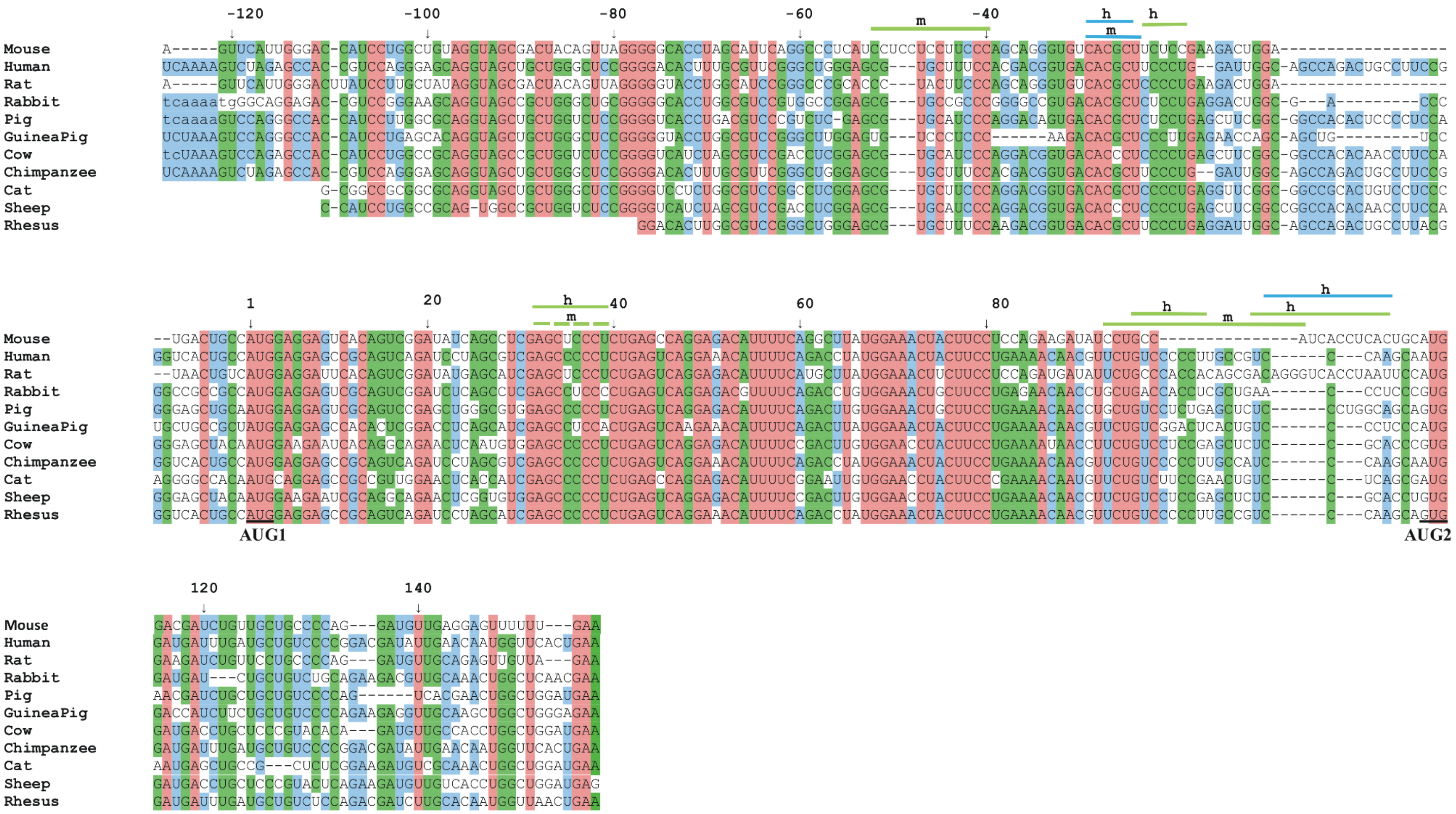
-120
-100
-80
-60
-40
m
h
h
m
Mouse
Human
Rat
Rabbit
Pig
GuineaPig
Cow
Chimpanzee
Cat
Sheep
Rhesus
1
20
40
60
80
AUG1
AUG2
120
140

Figure S6. Alignment of the 5′-terminal regions of p53 mRNA sequences derived from eleven different species (modified from (Szpotkowska et al. 2019; Figure 2)) with binding sites of hnRNP K and PCBP2 proteins. Binding sites of hnRNP K (blue lines) and PCBP2 (green lines) are marked along the sequences for mouse and human p53 mRNA (denoted with 'm' and 'h' letters, respectively). Capital letters symbolize mRNA sequence (derived from GeneBank) and lowercase letters indicate *p53* gene sequence (obtained from Ensembl) of the region homological to the longest identified mouse transcript. Translation initiation codons AUG1 and AUG2 are depicted. Alignment is colored according to the percentage of sequence conservation (red, 100%; green, 80% – 99%; blue, 60% – 79%).
